# Earthquake-related stressors associated with suicidality, depression, anxiety and post-traumatic stress in adolescents from Muisne after the earthquake 2016 in Ecuador

**DOI:** 10.1186/s12888-020-02759-x

**Published:** 2020-07-02

**Authors:** Rebekka M. F. Gerstner, Fernando Lara-Lara, Eduardo Vasconez, Ginés Viscor, Juan D. Jarrin, Esteban Ortiz-Prado

**Affiliations:** 1 Department of Psico-etichs, Pontificia Universidad Católica del Ecuador, sede PUCE Santo Domingo, Santo Domingo de los Tsachilas, Ecuador; 2 OneHealth Research Group, Faculty of Medicine, Universidad De Las Americas, Calle de los Colimes y Avenida De los Granados, 170137 Quito, Ecuador; 3 Faculty of Medicine, Universidad Central del Ecuador, Quito, Ecuador; 4 Physiology Section, Department of Cell Biology, Physiology and Immunology, Universitat de Barcelona, Barcelona, Spain

**Keywords:** Suicidality, Depression, Anxiety, PTSD, Ecuador earthquake

## Abstract

**Background:**

The Ecuadorian earthquake in April 16th was the second strongest and deadliest in 2016 worldwide, with approximately one million people affected. In this paper, we analyzed the psychological impact and the relationship between mental health events and various earthquake-related stressors related to the earthquake, 9 months after the event.

**Methods:**

We conducted an analytical cross-sectional study, applying an anonymous survey to 316 adolescents (13–19 years old) from Muisne, Ecuador. Suicidal tendency, depression, anxiety and post-traumatic stress (PTSD) were evaluated via the Child PTSD Symptom Scale (CPSS), *Spence Children’s Anxiety Scale*, *Okasha Suicidality Scale*, and the Center for Epidemiologic Studies Depression Scale (CES-D) and the adapted seven-questions earthquake-related stressors survey.

**Results:**

We found a high prevalence of suicidal ideations and behavior, posttraumatic stress, depression and anxiety compared to international studies. Even though adolescents currently living in shelters had higher levels of anxiety, their suicidal tendency was significantly lower than those living in their own or their relatives’ home. Finally, the earthquake-related stressors were not associated with suicidality and mental health events, with the exception of economic damage suffered by the family.

**Conclusions:**

High levels of depression, post-traumatic stress and anxiety among high-school students were found, especially among those who have suffered serious economic damage.

The economic impact in their families and high unemployment rates among their parents seems to be related to lack of hope and favorable perspectives for their future, situation that might lead to lead to emotional disturbances and psychological disorders. Although prolonged homelessness experience in shelters may be a stressful occurrence, might also be related with spiritual growth among adolescents, and may work as a protective factor against suicidal ideations and attempts.

## Background

On April 16, 2016 (16A) Ecuador suffered one of the strongest earthquakes in its history, with a magnitude of 7.8 on the Richter scale, which resulted in 663 fatalities, approximately 30,000 people were evicted and about a million people were indirectly affected [1].

Witnessing a natural disaster is an experience that affects people deeply, resulting in anxiety and stress [2]. These reactions, which at the beginning appear to be a normal response can become a serious mental health problem when the level of stress and anxiety experienced at the beginning does not decrease over time [3]. While most people are resilient to the impact of an event of this severity, about 30% of those affected develop mental-health disorders [4, 5]. According to these investigations, the most vulnerable group for the psychological impact of the disaster corresponds to young people from developing countries. There is much evidence that many adolescents who experience a natural catastrophe develop symptoms of posttraumatic stress [6], depressive disorders [7] and anxiety [8].

Post-traumatic Stress Disorder (PTSD) according to the American Psychological Association appears after a traumatic experience —the threat or experience of injury or death of one’s own or of a very close person— and lasts for a period greater than 30 days [9]. On the contrary, anxiety disorders are fears that may, in some cases, be directly caused by a traumatic event, but they do not necessarily have to be associated with an experience like this. The most common anxious disorders after a catastrophe or disaster experience are generalized anxiety and panic disorders [4]. In addition to posttraumatic stress and anxiety, depression is another common psychological disorder among adolescents as a result of severe cataclysms [10]. Some of the factors that are associated with emotional stress within the population depend on the perception of external help, especially for those victims who are evicted and placed in temporary shelters, because the concern about an unfair distribution of resources, causes discomfort and additional stress in the community [11].

### Natural disasters and suicide

The influence of a natural disaster on the risk of committing suicide has been investigated before [12–15]. While some research suggest a reduction in suicide risk during the first few months or years after the disaster [16, 17], other studies suggest an increase in suicide related deaths after such events [13, 18].

There are some determinants that can explain the increase or decrease in suicidal behavior of the population in regions hit by natural disasters such as: the severity of the disaster, social cohesion among the affected people and the support received by regional or national governments as well as from international NGO’s [19, 20]. Kõlves indicate that, in addition, the period of study after the disaster is essential [12]. In this sense, some authors differentiate the following stages: heroic, honeymoon, disappointment and reorganization (restoration) [21].

The heroic phase is the first moment in which most of the fears, confusion and insecurity are experienced. It can last up to 1 week. The Heroic phase, in which adrenaline levels rise, the population is now gaining the ability to save themselves and their family, help others and organize help from strangers. In this period, altruistic attitudes are usually observed in the majority of those affected as well as in nearby populations that were not affected. The Honeymoon phase, in which optimism prevails and hopes that everything will be the same (or even better) as before the disaster [21]. Normally it lasts between 2 and 4 weeks. The altruistic attitude persists in the affected community and throughout the country. According to the authors, in the first emotional phase after the earthquake - called the honeymoon phase, a stage where the suicidal behaviors in the population tend to decrease, and to increase later in the subsequent phases. The stage of disappointment is the moment in which those affected are contacted with the harsh reality they are confronting; they perceive that there are not enough resources for everyone and that they are often unfairly distributed. This is the stage in which there is the greatest potential for the development of psychological disorders and usually begins from the second or third month after the disaster and lasts up to 36 months. The last stage is the one of the destabilizations in which the population adapts to its reality and the lost thing is reconstructed and recovered [21].

In addition, although the population as a whole is affected by a natural disaster, WHO insists on the concern for especially vulnerable groups such as children and adolescents, due to their dependency relationship with adults and its specific state of development [4]. This age group, being socially and economically dependent on its family and due to its conflictive period of life, has a special risk for the development of psychological disorders and suicidal tendencies after a natural disaster [22]. As for the earthquake in Ecuador, although the province of Manabí received the greatest impact in relation to human and material losses, Esmeraldas province also reported injuries and significant damage to buildings and infrastructure. The most affected canton in the latter province is Muisne, where the highest number of injured and affected buildings were registered [1]. This area in the Ecuadorian coast was also the epicenter of several aftershocks greater than 6.0 on the Richter scale, between April and July 2016 [1]**.** According to one report published by Garcia et al. in 2016, at least 7000 refugees were still looking for official shelters 6 months after the catastrophe [1].

Given the situation described, this research aims to determine the prevalence of suicidal tendencies of the adolescent student population in Muisne, Esmeraldas 9 months after the earthquake. This time gap fits the disappointment phase, in which mental health issues related to the earthquake generally start [21]. In the same way, we aim to measure if the prevalence of mental health events such as depression, anxiety and post-traumatic stress and suicidal tendency differs according to exposure to different earthquake-related stressors, including prolonged lodging in shelters or physical, material and economic damages suffered after the earthquake. Finally, we aim to evaluate the impact of depression, anxiety, posttraumatic stress disorder and earthquake related stressors in suicidal tendency among local adolescents that experienced the natural disaster at first hand.

## Methods

### Settings

This study was completed in Muisne, a coastal town in the province of Esmeraldas in Ecuador. Ecuador is a country located in Southamerica sharing borders with Peru and Colombia with 16,623.000 inhabitants. The Esmeraldas province has 7 cantons including Muisne, being this canton, a beach town located an hour and a half from the capital of the province.

The coastal town of Muisne runs along 8 km of coast and has an estimated population of 28, 047 inhabitants, being 50.03% women and 49,97% men, most of them afro-Ecuadorians.

This part of the country was heavily affected by the 2016 earthquake and subsequent aftershocks.

### Variables

Socio-demographic variables such as age, sex, and level of education were analyzed, as well as the variables of interest regarding earthquake-related-stressors, Post-traumatic Stress Disorder, anxiety, depression and suicidal risk*.*

### Post-traumatic stress disorder symptoms

The Child PTSD Symptom Scale (CPSS) was created based on previously published studies [23–26], to assess the presence of PTSD symptoms in children and adolescents between 8 and 18 years in terms of the last 2 weeks.

In the present study, we used the Spanish version translated by the Chilean researchers Bustos, Rincón and Aedo, instrument validated in 2009 and adapted to the Ecuadorian context and the situation [27]. The answers may vary from: 0 (never or only once), 1 (occasionally), 2 (half the time) and 3 (almost always). For the present study, the 17 items of its original version were used. The possible scores vary from 0 to 51 and the cut-off point was established at 24 points [23]. The scale showed high internal consistency and good test-retest reliability in its original version [23] as in the Chilean adaptation [27].

### Anxiety

The Spence Children’s Anxiety Scale, (SCAS) was used to evaluate the presence of different anxiety disorders among children and adolescents [28]. The SCAS provides a description of the severity of specific anxiety symptoms for both children and young adolescents [29] and is characterized by measuring the specifications of anxiety disorders in children and adolescents. For this work, a validated version in Spanish was taken [30, 31], with proven reliability and validity [32, 33]**.**

In order to shorten the questionnaire, we used only three subscales (21 items) of original 38 items, which evaluated the types of anxiety directly linked to natural disasters: panic attacks, separation anxiety and generalized anxiety disorders. The internal consistency of the mentioned subscales varies from 0.81 (panic attacks) to 0.75 (separation anxiety) and 0.67 (generalized anxiety); Cronbach’s alpha for the total scale is 0.89. Since there is no cut-off point for these three subscales in this study, the degree of affectation was determined considering one standard deviation from the media that in the present case was 35 points.

### Prevalence of depression

The Center for Epidemiologic Studies Depression Scale (CES-D) scale was designed to determine prevalence of depression in adolescents and adults in a general population [34]. The inventory evaluates the presence of 20 depressive symptoms during the last week, with a response format of: No day = 0, 1 to 3 days = 1, 4 to 6 days = 2 and every day = 3.

The scale has been validated in Mexican and Chilean students [35], Colombians [31, 36] and Peruvians [37], with a good result in terms of reliability and validity. In the present study, the version used among Ecuadorian adolescents presents a Cronbach’s alpha of 0.81. We used the cut-off point of 24, methodology validated by Fuentealba et al., 2004 in a similar study among youths from Chile, in Southamerica [35].

### Suicidal risk

The Okasha Suicidality Scale was created by Okasha et al. in 1981 and was used to determine the risk of committing suicide within this community [38]. This scale measures suicide risk and consists of 4 items: 1. Have you ever thought that life is not worth it? 2. Have you ever wanted to be dead? 3. Have you ever thought about ending your life? 4. Have you attempted suicide?

The first three items have a 0 to 3 format as a response option (never, almost never, sometimes, many times). In the present study a fifth item was added: Have you made any plans to take your life?, since the plan plays an important role for the assessment of suicide risk [39]. In order to evaluate the impact of the earthquake, the scale was adapted, asking for suicide risk only in the last 9 months (time since the earthquake occurred).

The original scale was translated into Spanish and validated by Salvo et al. 2009 in a teenage population in Chile with an internal consistency of 0.89 [40]. The cut-off point for the subscale of suicidal ideations, to identify people with high suicidal tendencies, was set at 5 points [40].

### Earthquake-related stressors

An adapted seven-questions survey based on the study conducted by Díaz, Quintana and Vogel was used to explore the earthquake-related stressors [41]. The questionnaire contained seven items that were intended to identify the earthquake-related stressors that might be linked to mental health problems or suicide risk after an earthquake. The questions were intended to explore the timeline related to the earthquake, the degree of damage in houses and the consecutive economic losses, the current housing and the housing just after the earthquake, the physical damages and life losses affecting their selves and their relatives and the psychological support (if any) that was available just after the disaster.

The social determinants (earth-quake related stressors) were dichotomized. We decided to do so because the items included several categories, starting from very mild affections to seriously affecting stressors. Thus, we decided to purposefully dichotomized them in no affection and affections. After doing this, we were able to complete binomial analysis using a linear regression model.

### Data quality and validation

Missing data was replaced by the average of each participant, value produced after calculating the mean after completing the scale. For reasons of accuracy, no missing was replaced if they represented more than 20% of the scale.

### Statistical analysis

For the two groups comparisons we used a t-test in order to compare means obtained in the measured scales (suicidal tendency and other mental health events between the samples).

In terms of the earthquake-related stressors we dichotomized them according to the presence or not of any effect (0 if the damage has been slight or nonexistent and 1 if it has been serious). To contrast the dichotomized and the demographic variables (gender and age) a Pearson Chi-square test was used. A correlation and a linear regression analyses were run using the sum of all the earthquake-related stressors in order to estimate the impact of various stressors on mental health and suicidality tendencies.

All statistical analysis accepted significance with an alpha level of 0.05%. The statistical analysis was completed using the IBM SPSS statistics version 24.0.

## Results

### General results

Of the total student sample, 55% had been found on the island side of Muisne during the earthquake, 27% were located on the mainland of Muisne parish and the remaining 17% in some other part of Ecuador.

### Descriptive statistical analysis

Table 1 show several descriptive statistical data such as: simple size, mean values, standard deviation, significance level (alpha value) and internal consistency for each of the main variables collected in this study: suicidal tendency, depression, post-traumatic stress disorder symptoms and anxiety.

**Table 1:** Descriptive statistics

Variable	n	Mean	SD	α	Potential	Real	Asymmetry
Suicidal tendency	255	2.78	3.11	0.83	0–15	0–12	1.03
Depression	283	20.03	9.39	0.79	0–60	2–49	0.45
PTSD	301	19.37	10.10	0.85	0–51	0–44	0.25
Anxiety	287	23.84	11.01	0.89	0–63	1–60	0.31

Note: *SD* standard deviation, *α* significance level, *PTSD* Posttraumatic Stress Disorders

### Suicidal tendency

Less than a half of the students who participated, indicated that they had never had suicidal ideations. Around 30% declared that during the last 9 months they thought sometimes or many times that life is not worth it and wished they were dead. Also, 20% thought sometimes or many times about ending their lives, and 13% on several occasions have made concrete plans to commit suicide.

Female adolescents suffered from significantly higher levels of suicidal ideations than men, being the most frequent expression in them the “desire to be dead”, with almost % of adolescent girls who claim to have wished at least sometimes during the last 9 months being dead, compared to 44% of boys (*X*^2^ (1) =13.71, *p* < .001). In addition, 49% (women) and 34% (men) of the participants indicated that they had ever thought that life is not worth it (*X*^2^ (1) =7.10, *p* = .008). In the same way, one in five men claimed to have thought about ending their lives, compared to around 44% of female adolescents (*X*^2^ (1) =17.99, *p* < .001). There were gender differences in suicide plans during the last 9 months. While the data indicate that 27% of women had made plans at some time, only 15% of men declared the same (*X*^2^ (1) = 7.93, *p* = .005).

### Suicide attempts

Regarding suicide attempts, 205 adolescents (65%) indicated that they had never attempted suicide, while 23 (7%) claimed to have tried once, 12 (4%) twice, 8 (3%) three times and 4 (1%) four or more times. Is important to remark that there was a high number of absence of answers to this question, because 21% (*n* = 64) refused to answer it.

Regarding gender differences in previous suicide attempts, 20% of women reported having tried at least once to take their own lives, compared to only 9% of men (*X*^2^ (1) =7.34, *p* = .009).

### Depression, anxiety and PTSD prevalence

Table 2 reports the prevalence of depression, posttraumatic stress and suicidal tendency for the total sample considering their age and gender. The prevalence of depression and post-traumatic stress is high, since approximately one third of the participants appear to be affected by at least one of these alterations. In addition, it is observed that women have significantly higher scores in the four variables compared to men.

**Table 2:** Prevalence of depression, anxiety, post-traumatic stress and suicidal tendency in the total sample and by gender

	Gender			
	Total (*n* = 300)	Men (*n* = 144)	Women (*n* = 156)	χ 2
**Depression (> 24)**	28.5%	19.9%	36.1%	9.37^**^
**Anxiety (> 35)**	15.2%	6%	21.8%	15.14^***^
**PTSD (> 24)**	33.2%	23.8%	42.2	10.97^***^
**Suicidal tendency (> 5)**	18.2%	11.9%	17.5%	8.01^***^

Note: *PTSD* Posttraumatic stress disorder, *n.s.* not significant

***: < 0.001; **:< 0.01; * < 0.05

Table 3 reports the ANOVA results of the effect that various earthquake-related stressors resulting from the disaster on the suicidal tendency of adolescents: depression, anxiety and post-traumatic stress. It is noteworthy that shelter accommodation (official or unofficial) has no effect on mental health events in the first stage after disaster.

**Table 3:** Social determinants and risk factors in relation to mental health events

	Depression		PTSD		Anxiety		Suicidal tendency	
	Mean ± SD	P	Mean ± SD	p	Mean ± SD	p	Mean ± SD	p
**Accommodation after earthquake**								
Home	20.65 ± 9.28	0.202	19.32 ± 10.52	0.888	23.14 ± 11.40	0.156	3.02 ± 3.21	0.129
Shelter	19.13 ± 9.63		19.50 ± 9.47		25.07 ± 10.37		2.40 ± 2.95	
**Current accommodation**								
Home	19.71 ± 9.11	0.089	18.91 ± 10.12	0.056	23.20 ± 10.99	0.017	2.90 ± 3.22	0.019
Shelter	22.72 ± 11.27		22.33 ± 9.67		27.84 ± 10.58		1.84 ± 1.93	
**Damages on family housing and properties**								
Severe damage	19.92 ± 9.18	0.671	19.11 ± 10.36	0.383	23.11 ± 11.27	0.082	2.82 ± 3.14	0.840
None or small	20.46 ± 9.89		20.28 ± 9.31		25.57 ± 10.20		2.73 ± 3.08	
**Economic damage**								
None or small	18.86 ± 8.94	0.000	17.98 ± 9.86	0.000	22.78 ± 10.92	0.001	2.78 ± 3.08	0.762
Job loss	24.10 ± 9.92		24.30 ± 9.38		27.65 ± 10.49		2.64 ± 3.25	
**Physical damages and severity**								
None or minor	19.78 ± 9.21	0.143	19.07 ± 9.83	0.161	23.51 ± 10.75	0.094	2.73 ± 3.10	0.354
Serious or death	22.78 ± 11.03		22.76 ± 12.46		27.36 ± 13.17		3.42 ± 3.27	
**Psychological help or counselling received**								
No help	19.69 ± 9.07	0.294	19.16 ± 10.00	0.408	23.84 ± 11.28	0.923	2.74 ± 3.13	0.549
At least once	20.92 ± 9.73		20.17 ± 10.26		23.97 ± 10.61		2.99 ± 3.10	

#### Regression analysis

Lineal regression was carried out to test the predictive power of depression, anxiety, PTSD and earthquake related stressors for suicidal tendency (Table 4). These factors explain altogether 25% of variance in suicidality, with highest beta coefficients for depression (β = .30, *p* = .003), generalized anxiety disorder (β = .20, *p* = .013), gender (β = .20, *p* = .003), posttraumatic stress disorder (β = .19, *p* = .025) and at least earthquake related stressors (β = .16, *p* = .013).

**Table 4:** Lineal model to predict suicide risk, with 95% bias corrected and accelerated confidence intervals reported in parentheses. Confidence intervals and standard errors based on 1000 bootstrap samples

	b	SE B	β	p
**Constant**	−2.749 (−6.5, .755)	1.812		.126
**Gender (0 = man. 1 = woman)**	**1.352 (.436, 2.263)**	**.467**	**.197**	**.003**
**Age**	.105 (−.100, .317)	.108	.058	.330
**Depression**	**.116 (.050, .190)**	**.037**	**.298**	**.003**
**Generalized anxiety disorder**	**.187 (.053, .323)**	**.069**	**.203**	**.013**
**Separation anxiety**	−.093 (−.260, .071)	.079	−.094	.234
**Panic disorder**	−.074 (−.214, .043)	.067	−.112	.266
**Posttraumatic stress disorder**	**.066 (.006, .131)**	**.030**	**.185**	**.025**
**Earthquake related stressors**	**−.483 (−.866, −.074)**	**.192**	**−.155**	**.013**

Note: bootstrap results are based on 1000 bootstrap samples

The three subscales were analyzed separately: panic attacks, separation anxiety, and generalized anxiety disorders.

## Discussion

We have found a high prevalence of suicidal ideations, plans and attempts in adolescents in Muisne, and important differences are distinguished in terms of their frequency between men and women.

Regarding suicide attempts, 15% of adolescents indicated that they attempted suicide (21% of women and 9% of men). These are higher figures in comparison with those reported in other investigations. Most studies report a low percent varying in a range between 4 to 7% [12]. However, a study conducted in India after a cyclone confirms the prevalence of similar suicide attempts such as those found at 12.6% [42]. The highest numbers of suicide attempts in the Ecuadorian sample and in the population of India are possibly related to both being regions with high rates of consummate suicide [43], even before the natural disaster suffered by these countries. There could, therefore, be an underlying socio-cultural trend and perception of suicidal behavior that together with an additional stressful event (natural disaster) leads to a high number of attempts.

We found that one third of adolescents have symptoms of PTSD and exceed the cut-off point of the instrument used. This proportion is higher than those reported in the study by Díaz et al. [41], which was conducted with adolescents of similar ages in regions affected by a tsunami in Chile (33% compared to 20%). The prevalence of post-traumatic stress is probably related to the severity of the traumatizing event, as Muisne students report a greater degree of physical damage (severe injuries and family losses) and property damage than Chilean adolescents. Therefore, the impact of the disaster may explain a greater impact on the mental health of the students.

Regarding the levels of depression (29%) and anxiety (15%) found, they differ slightly from those reported in the above mentioned study by Díaz et al. [41], with 30 and 14% respectively. Different studies carried out in other regions of the world yield figures comparable to those of Muisne. Thus, three studies that studied the prevalence of depression in groups of adolescents between 6 and 12 months after an earthquake show a prevalence of 31% in Turkey [44] and between 25 to 37% in China [45, 46]. Regarding anxiety, the data seem discordant with prevalence’s of 17% [47] to 40% in China [46], compared to 15% in this study.

### Earthquake-related stressors and their relationship with suicidal tendency, depression, anxiety and post-traumatic stress

Some earthquake-related stressors such as physical damage or loss of family members, damage to property or other material losses, accommodation in shelters, and economic losses in the family, and their relationship with mental health events were evaluated in the present study.

Although similar percentages to our findings are reported in a study with adolescents in the city of Concepción (Chile) after the telluric movement of 8.8 [41] in regard to economic losses, property damage was much more widespread in the present case (30%) compared to that occurred in Chile (14%), as well as physical damages to family members (6 to 3.3%) and family losses (2.4% in Muisne vs. 1% in the Chilean case).

Only the adolescents who were living longer than 9 months in temporal shelters had significantly higher levels of anxiety compared to those living in their own or relatives’ homes. After spending several months in shelters, the risk of developing anxiety disorders is high [48]. Uncertainty in young adults generates chronic stress which activates our basic instincts for survival [49]. In this situation, the frontal cortex, the amygdala, the hypothalamus and the suprarenal glands are responsible for producing heightened effects in cortisol and adrenaline [50]. These are directly linked to physical and cognitive symptoms which lead to anxiety disorders. However, because of constant peer and social support possibly depressive disorders were reduced.

On the other hand, the suicidal tendency of adolescents currently living in shelters was significantly lower than those living in their own home or in the home of relatives. In addition, there are no significant differences between the level of depression and post-traumatic stress when comparing the type of accommodation after the earthquake or at the time in which the study was performed.

These factors can be considered as earthquake-related stressors that possibly explain the high prevalence of emotional disturbances in our studied population. When measuring the relationship between earthquake-related stressors and mental health events of adolescents, contradictory results were obtained. On the one hand, this study shows that large economic (or job) losses in the family are associated with higher levels of depression, anxiety and post-traumatic stress; but at the same time, it cannot be demonstrated that there are significantly higher suicidal tendencies in our sample. This result contrasts with that obtained by Kar [51] in a study with those affected by a cyclone in India, where one of the best predictors for high suicidality was job loss and other psychosocial factors. In this sense, the loss of work or business can be considered as a risk factors for a suicidal tendency, only in those directly affected, but not in those indirectly affected, that is the family; however, this rule cannot be extended to high levels of depression, anxiety and PTSD.

There were no statistically significant differences in any of the mental health factors related to housing damage, physical damage and even the death of close relatives. The loss of home, the stay in shelters and the physical damages or even the death of relatives, are associated with mental health events (including the suicidal tendency), but this relationship is not statistically significant except for the young people staying in shelters, in which high levels of anxiety are found. This result partially confirms those described in previous studies in Turkey and Taiwan [52, 53] in which the damage or loss of the property was positively related to depression and suicidal ideation.

Our results demonstrated that the levels of depression, anxiety and post-traumatic stress of adolescents whose parents have had a serious economic loss or even job loss, are significantly higher than those who have not suffered economic losses (or those were temporary). Our shows clearly that the suicidal tendency is not higher in students whose families have had greater economic losses, despite the fact that scores on depression, anxiety and post-traumatic stress turn out to be much higher.

On the other hand, it is suggestive that the adolescents who remained until January 2017 in the shelters showed higher levels of post-traumatic stress, depression and anxiety, but at the same time, lower suicidal tendencies than those who continued to live at home. This apparent contradiction is explained in a longitudinal investigation conducted before and after Hurricane Katrina in the United States [54]. Although significantly higher prevalence of depression, anxiety and post-traumatic stress were found in the sample after the hurricane, suicidal ideation was lower compared to the measurement taken before the disaster. Additionally, some mediating factors were measured, and it was found that spiritual growth and an improvement in personal relationships (experiences framed during the disaster) probably reduced suicidal ideations [44]. In the same line, Yu et al. measured suicidal ideations in adolescents before and after an earthquake in China, with lower prevalence (10.6%) after the event as compared to the initially reported level (19.3%), probably as a result of post-traumatic increase as suggested by these authors.

It is tempting to speculate that the prolonged experience in shelters stimulates the spiritual growth in adolescents, since even being victims of these adverse circumstances and suffering high levels of depression, anxiety and PTSD, the relationship with the other affected and helping volunteers may work as a factor of protection against suicidal ideations and attempts.

In turn, the economic damage or loss of work of parents seems to directly impact family life, affecting hope and favorable perspectives for their future, leading to emotional disturbances and psychological disorders.

High levels of depression, post-traumatic stress and anxiety in high school students in this area, especially in those who have suffered serious economic damage, may also possibly be due to factors inherent to the political and social conditions in this area.

During the reconstruction phase (9 months after the disaster) the population generally goes through a period marked by disappointment and hopelessness, especially if there is a perception that the government is favoring other areas or that reconstruction materials are being distributed unfairly. However, this circumstance has not been addressed in this paper although there are abundant references in the media.

## Conclusions

High levels of depression, post-traumatic stress and anxiety in high school students, especially in those who have suffered serious economic damage, may possibly be due to factors inherent to the political and social conditions in this area.

In turn, the economic damage or loss of work of parents seems to directly impact family life, affecting hope and favorable perspectives for their future, leading to emotional disturbances and psychological disorders. Whereas the prolonged experience in shelters may be a stressful experience, it also possible stimulates the spiritual growth in adolescents, and may work as a factor of protection against suicidal ideations and attempts.

## Limitations

It is necessary to mention the transverse character of this investigation as much as it does not allow causal inferences between the variables. Also, the prevalence of mental health events prior to the earthquake is unknown, so it is not possible to make comparisons with pre-disaster prevalence. Studies on the prevalence of suicidal ideations and attempts in adolescents before the earthquake in Muisne are scarce or nonexistent, however some previous studies indicate that adolescents in Ecuador in general have high levels of depression, ideation and suicidal attempts (Author, 2012); Jaramillo, 2009; Author, 2018).

In general, the methodology used involves other difficulties, since the collection of data through questionnaires can lead to reading and comprehension errors, and consequently the results must be interpreted with some caution.

Finally, for logistical reasons, this study was carried out at the end of the school year, which could be understood as a time of high intellectual stress for high school students, so this factor may have influenced their mental health.

## Data Availability

All data by including sensitive information are not publicly available, however, under a rational requirement, the data can be shared by writing a request to the following email: remgerstner@gmail.com

## Abbreviations

PTSD: Post-traumatic stress
CPSS: Child PTSD Symptom
NGO: Nongovernmental organization
SCAS: The Spence Children’s Anxiety Scale
CES-D: The Center for Epidemiologic Studies Depression Scale
ANOVA: Analysis of variance
