# Correction to: Earthquake-related stressors associated with suicidality, depression, anxiety and post-traumatic stress in adolescents from Muisne after the earthquake 2016 in Ecuador

**DOI:** 10.1186/s12888-021-03238-7

**Published:** 2021-05-05

**Authors:** Rebekka M. F. Gerstner, Fernando Lara-Lara, Eduardo Vasconez, Ginés Viscor, Juan D. Jarrin, Esteban Ortiz-Prado

**Affiliations:** 1 Department of Psico-etichs, Pontificia Universidad Católica del Ecuador, sede PUCE Santo Domingo, Santo Domingo de los Tsachilas, Ecuador; 2 OneHealth Research Group, Faculty of Medicine, Universidad De Las Americas, Calle de los Colimes y Avenida De los Granados, 170137 Quito, Ecuador; 3 Physiology Section, Department of Cell Biology, Physiology and Immunology, Universitat de Barcelona, Barcelona, Spain; 4 Faculty of Medicine, Universidad Central del Ecuador, Quito, Ecuador

**Correction to: BMC Psychiatry 20, 347 (2020)**

**https://doi.org/10.1186/s12888-020-02759-x**

Following the publication of the original article [1], the authors identified an error in the affiliation of Dr. Ginés Viscor. The correct affiliation is given below.

Physiology Section, Department of Cell Biology, Physiology and Immunology, Universitat de Barcelona, Barcelona, Spain.

The original article [1] has been corrected.
